# Special Issue “Role of NRF2 in Disease: Novel Molecular Mechanisms and Therapeutic Approaches”

**DOI:** 10.3390/biom11020202

**Published:** 2021-02-02

**Authors:** Isabel Lastres-Becker

**Affiliations:** 1 Instituto de Investigaciones Biomédicas Alberto Sols (IIBM), UAM-CSIC, 28029 Madrid, Spain; ilbecker@iib.uam.es; 2 Departamento de Bioquímica, Facultad de Medicina, Universidad Autónoma de Madrid (UAM), 28029 Madrid, Spain; 3 Instituto de Investigación del Hospital Universitario de La Paz (IdiPAZ), 28047 Madrid, Spain; 4 Centro de Investigación Biomédica en Red Sobre Enfermedades Neurodegenerativas (CIBERNED), ISCIII, 28013 Madrid, Spain

This Special Issue on NRF2 (https://www.mdpi.com/journal/biomolecules/special_issues/role_nrf2) highlights the relevance of this transcription factor in a broad spectrum of diseases such as cancer, neurodegenerative disorders, diabetes, sepsis, skin diseases, and arsenic toxicity.

Regarding the implication of the NRF2/KEAP1 pathway in tumor metabolism, Panieri et al. [1] reviewed how metabolic reprogramming is tightly interconnected with the regulation of redox homeostasis and suggested that alterations in NRF2 signaling could be a therapeutic strategy to fight against solid and hematologic cancers. More specifically, in colorectal cancer, Torrente et al. [2] demonstrated that the NRF2 axis is upregulated in colorectal tumors and that high levels of nuclear NRF2 correlate with poor patient prognosis. Furthermore, they identified that a specific aurora kinase inhibitor (AT9283) is capable of selectively killing cancer cells that have high levels of NRF2 as a result of either genetic or pharmacological activation.

In the field of neurodegenerative diseases, the relevance of NRF2 has been widely demonstrated. In this context, in this Special Issue, there are two reviews focused on DNA repeat expansion disorders [3] and on the key mechanisms implicated in NRF2 regulation and their potential use as targets for the development of new therapies for neurodegenerative diseases [4]. One of the main hallmarks of neurodegeneration and other pathologies is the presence of chronic inflammation. In this context, Henning et al. reviewed the molecular mechanisms triggering the therapeutic potential of dimethyl fumarate (DMF) and other NRF2 activators. They indicated that electrophiles also target other inflammation-associated pathways, such as the transcription factor NF-κB and the inflammasomes, and how they are interconnected with NRF2 [5].

In this Special Issue, there are also works related to other fields; for example, Teena et al. demonstrated that epigenetic changes such as the upregulation of several histone deacetylases (HDAC), such as HDAC1, 3, 4, and 11 and SIRT3; downregulation of HDAC2, 8, SIRT1, SIRT2, SIRT6, and SIRT7; and their association with the NRF2 axis and inflammatory markers are indicative of their implication in the pathogenesis of diabetic foot ulcers [6]. Moreover, the role of NRF2 is most significant in diseases associated with the generation of elevated concentrations of reactive oxygen species (ROS), leading to tissue and organ damage such as in sepsis. Gunne et al. summarized the recent studies relating NRF2 and sepsis patients in critical care [7]. Exposure to arsenic induces the production of intracellular ROS, which is implicated in multiple changes in cell behavior associated with signaling pathways and epigenetic modifications or triggering direct oxidative injury to molecules. Hu et al. reviewed the pathways involved in arsenic-induced redox imbalance as well as the current findings on treatment strategies using antioxidant molecules [8]. 

In general, this Special Issue demonstrates the relevance of the NRF2 factor in a wide spectrum of pathologies and how its pharmacological modulation can be used as a promising therapeutic target.
